# Transcranial Magnetic Stimulation over the Left Inferior Parietal Lobule Facilitates Early-Stage Processing During Natural Chinese–English Bilingual Reading

**DOI:** 10.3390/brainsci16050530

**Published:** 2026-05-17

**Authors:** Junjie Wu, Ruoling Hang, Pingping Xin, Guoli Yan, Chanyuan Gu, Luyao Chen

**Affiliations:** 1 Faculty of Psychology, Tianjin Normal University, Tianjin 300387, China; psywujunjie@tjnu.edu.cn (J.W.); 2230130424@stu.tjnu.edu.cn (R.H.); psyxpp@163.com (P.X.); yanguoli@tjnu.edu.cn (G.Y.); 2 Key Research Base of Humanities and Social Sciences of the Ministry of Education, Academy of Psychology and Behavior, Tianjin Normal University, Tianjin 300387, China; 3 Tianjin Key Laboratory of Student Mental Health and Intelligence Assessment, Tianjin 300387, China; 4 Department of Linguistics, Faculty of Medicine, Health and Human Sciences, Macquarie University, Sydney, NSW 2109, Australia; 5 Department of Language Science and Technology, Faculty of Humanities, Hong Kong Polytechnic University, Hong Kong 999077, China; chan-yuan.gu@connect.polyu.hk; 6 Department of Neuropsychology, Max Planck Institute for Human Cognitive and Brain Sciences, 04103 Leipzig, Germany; 7 Max Planck Partner Group, School of International Chinese Language Education, Beijing Normal University, Beijing 100875, China

**Keywords:** bilinguals, natural reading, eye-tracking, TMS, left inferior parietal lobule

## Abstract

**Background:** Proficient second language (L2) reading relies on complex neurocognitive processes. Neuroimaging studies have identified key brain regions recruited during L2 reading, including the left inferior parietal lobule (LIPL) and the calcarine cortex (CAL). The LIPL has been suggested to be involved in phonological decoding during L2 reading, whereas the CAL has been implicated in early-stage visual processing. However, given the correlational nature of neuroimaging techniques, it remains unclear whether these regions play causal roles in L2 reading or are merely epiphenomenal. **Methods:** To address this issue, the present study used transcranial magnetic stimulation (TMS) to modulate neural activity in these regions and eye-tracking technology to assess subsequent reading performance in Chinese–English bilinguals. Specifically, ninety-seven participants were randomly assigned to one of three offline TMS conditions: LIPL, CAL or vertex (as a control site) stimulation, after which they performed a natural sentence reading task in English. **Results:** The results showed that, compared to the control condition, TMS over the LIPL significantly reduced first fixation duration, whereas no significant effects emerged on gaze duration, regression path reading time, or total reading time. TMS over the CAL produced no significant effects on any eye-movement measures. **Conclusions:** These findings suggest that the LIPL plays a causal role in L2 reading for early-stage lexical processing through phonological decoding. Overall, this study is the first to employ TMS and eye-tracking to investigate the neural mechanisms underlying natural L2 reading.

## 1. Introduction

Reading in a second language (L2) places greater cognitive demand than reading in a first language (L1) [1]. Compared to native readers, L2 readers tend to exhibit longer fixation durations, slower reading speeds, and lower word-skipping rates during reading [2,3]. Neuroimaging studies have demonstrated that both L1 and L2 reading engage a left-lateralized language network encompassing frontal, temporoparietal, and ventral occipitotemporal regions [4,5,6]. Beyond this network, activity in the calcarine cortex (CAL), part of the primary visual cortex, has been linked to reading fluency [7]. Despite substantial overlap between L1 and L2 reading, the left inferior parietal lobule (LIPL) shows greater activation during L2 reading, reflecting increased demands on phonological processing [8,9]. However, such evidence remains correlational and thus provides limited insight into the causal roles of these regions in L2 reading. To address this limitation, the present study used transcranial magnetic stimulation (TMS) to modulate activity in the LIPL and CAL and examined the resulting effects on eye movements during natural sentence reading.

Eye tracking has long been used to investigate the real-time dynamics of reading [3,10,11]. Researchers have identified a range of eye-movement measures that capture individual differences in lexical access and reading efficiency [12,13]. For example, first fixation duration (FFD) records only the duration of the first fixation on a word during first-pass reading, reflecting initial orthographic encoding [14]. Gaze duration (GD) sums all fixation durations on a word during first-pass reading, capturing both lexical access difficulty and local reprocessing [10,14]. Regression path duration (RPD) and total reading time (TRT) have been reported to reflect later-stage processes such as semantic integration and syntactic analysis [14,15]. By recording the eye movements during English text reading among L2 readers, Kuperman et al. [3] found shorter fixation durations and higher skipping rates among readers with greater English proficiency. These findings suggest that these eye-movement indices are associated with L2 reading fluency.

A substantial body of neuroimaging research has elucidated the neural bases of reading processes in native readers. Evidence indicates that the cortical system supporting skilled reading comprises three principal subsystems: the left occipito-temporal region may subserve the automatized processing of visual word forms, the temporo-parietal region may be primarily responsible for orthography-to-phonology conversion, and the inferior/middle frontal gyrus region is associated with articulatory planning and phonological processing [4,5,16]. Prior to this network, early visual processing in the CAL provides the initial input to higher-order reading regions [17]. Henderson et al. [7] found that greater cortical surface area and volume of CAL were associated with shorter and less variable fixation durations. These findings suggest that structural properties of primary visual cortex predict individual differences in fixation duration, implicating early visual encoding efficiency as an important determinant of oculomotor control. A recent simultaneous eye tracking and functional magnetic resonance imaging (fMRI) study found that native English speakers exhibited significantly stronger CAL activation than Chinese–English bilinguals during natural reading, and that this activation correlated with multiple eye-movement measures ranging from early-stage measures (e.g., FFD) to later-stage measures (e.g., RPD) [18]. Given that CAL is regarded as a region primarily involved in early visual processing [7], and that neuroimaging methods are inherently correlational in nature, it remains unclear whether its association with later-stage eye-movement indices reflects a causal contribution or is merely epiphenomenal.

Studies investigating the neural mechanisms underlying L1 and L2 processing in bilinguals have generally found substantial overlap, both engaging a left-lateralized language network comprising frontal, temporoparietal, and ventral occipitotemporal regions [19,20,21,22]. Within this network, the LIPL has been associated with phonological processing in reading and is considered a key region for orthography-to-phonology conversion [9,23]. This function is particularly relevant in the context of L2 reading, where phonological demands are typically elevated [8,24]. Indeed, group differences in LIPL engagement have been consistently reported. For example, based on pathological studies of patients with parietal lesions, Pötzl [25] proposed that the LIPL is a critical region for regulating multilingual ability, referring to it as the so-called “language talent area,” as lesions in this region were associated with pathological language switching or selective impairment of second language ability. Mechelli et al. [26] found that gray matter density in the LIPL was greater in bilingual than monolingual individuals, and correlated positively with both early bilingual experience and second language proficiency. A meta-analysis by Li et al. [8] found that the LIPL showed significantly greater activation during L2 reading relative to L1 reading. However, existing evidence of neuroimaging is largely correlational, and whether LIPL activation causally drives L2 reading performance remains to be directly verified.

In recent years, transcranial magnetic stimulation (TMS) has emerged as a powerful tool for investigating causal brain function in both L1 and L2 processing [27,28,29,30,31,32,33,34]. It transiently modulates cortical excitability at targeted brain regions, allowing researchers to causally link regional neural activity to specific cognitive or behavioral processes [35,36]. The resulting aftereffects are thought to involve plasticity-like mechanisms, including changes in excitatory–inhibitory balance and processes related to N-methyl-D-aspartate (NMDA) receptors or gamma-aminobutyric acid (GABA) signaling [37,38,39]. For example, Arrington et al. [27] conducted a systematic review of 46 TMS studies of L1 reading and found that TMS modulates phonological processing after stimulation within the dorsal circuit (including the LIPL and the posterior superior temporal gyrus, STG). Wu et al. [40] applied TMS to the right inferior frontal gyrus (IFG) and assessed Chinese–English bilinguals’ performance in a picture-naming task. They found that stimulation over the right IFG significantly impaired language-switching performance, providing direct evidence for a causal role of the right IFG in bilingual language control. However, existing TMS studies of L2 processing have largely focused on lexical-level production or comprehension tasks, leaving it unclear what causal roles key cortical regions play in real-time processing during natural L2 reading.

The present study employed TMS to modulate activity in the LIPL and CAL among Chinese–English bilinguals reading English, followed by eye-tracking recording to capture real-time reading behavior. Specifically, participants were randomly assigned to one of the three groups: LIPL stimulation, CAL stimulation, or vertex stimulation as a control. After receiving TMS, participants performed a natural sentence reading task during which eye-tracking data were continuously collected. The study focused on TMS effects across eye-movement measures reflecting different processing stages: FFD as the earliest index of lexical recognition, GD reflecting slightly later word-level processing, and RPD and TRT indexing later-stage semantic integration. If the LIPL or CAL causally contributes to L2 reading, we predict that TMS over these regions should significantly modulate eye-movement measures relative to the control group. Conversely, if their activation is epiphenomenal, no significant differences would be expected.

## 2. Materials and Methods

### 2.1. Participants

Ninety-seven Chinese–English bilinguals from Tianjin Normal University volunteered to participate in the study (87 females, age = 21.40 ± 2.30 years). The participants learned English in primary school at the age of 7.71 ± 1.91 years. All of them had taken the College English Test Band 4 (CET-4; *M* = 510.47, *SD* = 44.53, maximum score = 710), a compulsory test measuring the English proficiency of undergraduate students in China. We further assessed their current English proficiency using the Oxford Placement Test (OPT; *M* = 34.80, *SD* = 5.07, maximum score = 50). L1 and L2 proficiency self-ratings were averaged across reading, writing, speaking, and listening skills (1 = very poor, 10 = very skilled). All participants were native Chinese speakers with normal or corrected-to-normal vision, aged 18 years or older, right-handed, and free of neurological conditions, seizure history, or TMS contraindications per established safety guidelines [35,36]. Participants were randomly assigned to one of three groups: the LIPL group, the CAL group, or the control group. The control group received TMS over the vertex. Demographic and language background characteristics of all three groups are summarized in Table 1. All participant characteristics were comparable across the LIPL, CAL, and control groups, with no significant differences between the experimental groups (LIPL and CAL) and the control group (all *ps* > 0.132). The study was approved by the Ethics Committee of Tianjin Normal University, and written informed consent was obtained from all participants prior to the experiment.

### 2.2. Materials

The experimental materials included five expository texts on science, technology, engineering, and mathematics (STEM), adapted from Follmer et al. [42]. Each article contained an average of 30 sentences and 306 words, maintaining a consistent linguistic style, as described in Ma et al. [43] regarding STEM texts and their psycholinguistic variables.

### 2.3. Procedure

**Figure 1:**
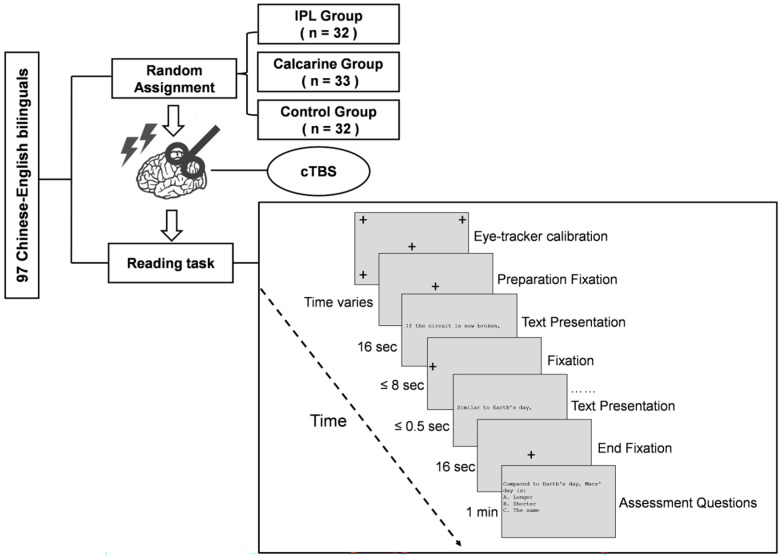
Experimental design and procedure. LIPL = the left inferior parietal lobule; CAL = the calcarine cortex; cTBS = continuous theta burst stimulation. Participants in the control group received TMS over the Vertex. The left panel illustrates the experimental design and group assignment, while the right panel displays the sequence and temporal structure of a single trial in the eye-tracking reading task.

This experiment used a single-blind, between-subjects design with one factor (stimulation site) varying across three levels: the LIPL, the CAL, and the control (receiving TMS over the vertex), see Figure 1. Participants were randomly assigned to groups and were unaware of their stimulation condition. TMS was targeted at the following Montreal Neurological Institute (MNI) coordinates: the LIPL (x = −40, y = −38, z = 48 [8]), the CAL (x = −12, y = −84, z = 0 [18]), and the vertex (x = 0, y = 0, z = 80 [41]).

Prior to the experiment, participants underwent structural scanning on a Siemens Prisma 3-T magnetic resonance imaging (MRI) scanner at Tianjin Normal University MRI Center for subsequent TMS neuronavigation. The images were obtained for co-registration with repeated time (TR) = 2530 ms, echo time (TE) = 2.98 ms, flip angle = 7°, field-of-view (FOV) = 256 × 256 mm^2^, matrix size = 256 × 256, resolution within slices = 1.0 × 1.0 mm, slice thickness = 1.0 mm, voxel size = 1 × 1 × 1 mm^3^, number of slices = 192, scanning duration ≈ 6 min.

To ensure real-time monitoring of stimulation accuracy, a frameless stereotaxic neuronavigation system (Localite GmbH, Bonn, Germany) was employed. Individual MRI scans were imported into the system and manually registered by localizing specific anatomical landmarks—the anterior and posterior commissures along with a reference point on the falx cerebri—to guarantee precise targeting. Subject-specific stimulation sites were defined via trajectory markers based on MNI coordinates. Prior to each TMS session, an MRI-guided neuronavigation procedure was employed to register the participant’s 3D anatomical scan to their physical head position in real space. A headband equipped with reflective markers was worn by the participant and tracked by the navigation system, enabling precise coil placement over the predetermined target site. Throughout the neuronavigation process, the coil’s orientation was continuously monitored to ensure the stimulation trajectory remained orthogonal to the skull surface.

Pulsed stimulation was delivered via a TMS stimulator (MagPro X100, MagVenture, Farum, Denmark) connected to a standard 70 mm figure-of-eight coil (MagVenture MCF-B65). Before administering TMS, participants’ resting motor threshold (RMT) was determined. This procedure involved applying single pulses of TMS over the motor cortex of the right hemisphere until clear motor-evoked potentials were detected in the first dorsal interosseous muscle in the left hand via electromyography. RMT was defined as the minimum machine output required to elicit a motor-evoked potential of at least 50 μV on at least 5 out of 10 consecutive trials. The stimulation intensity was 80% of the RMT [41]. We chose the continuous theta burst stimulation (cTBS) protocol, in which three pulses of stimulation are given at 50 Hz, repeated every 200 ms, and a 40 s train of uninterrupted TBS was given (600 pulses). Given that the neuromodulatory effects of cTBS typically last 30–50 min [44], whereas the reading task—consisting of five articles and corresponding comprehension questions—required approximately 60 min to complete, a supplementary cTBS application (using identical parameters) was administered before the fourth article. This was done to ensure the sustained neuromodulatory effects throughout the entire experimental session [45,46]. After the completion of both stimulation sessions, participants completed a TMS sensation questionnaire to assess whether the stimulation caused any discomfort. Given the violation of normality, non-parametric tests were used, specifically Kruskal–Wallis tests for the three-group comparison and Mann–Whitney U tests for pairwise comparisons. No significant differences were found across the three TMS groups (LIPL, CAL, and control) for any of the sensation items, including nausea, toothache, numbness, neck pain, face pain, ear pain, scalp pain, migraine, headache, nervousness/anxiety, vision problems, skin burn, fatigue, dizziness, thinking difficulties, speaking difficulties, and hearing difficulties. (all *p*s > 0.13). Pairwise comparisons against the control group also revealed no significant differences for either the LIPL or CAL group. Specifically, these exceptions included dizziness in the CAL compared with vertex condition (*p* = 0.064), and ear pain (*p* = 0.081) and nervousness/anxiety (*p* = 0.059) in the LIPL compared with control condition. Such discomfort is commonly reported in TMS research [47,48] and tends to resolve quickly following stimulation [49]. No participants withdrew from the experiment due to discomfort, and all confirmed feeling well before leaving the laboratory.

After receiving cTBS to modulate the target brain region, participants completed a natural sentence reading task while their eye movements were recorded. Eye movements of the participants were monitored using an Eye-Link 1000 Plus eye-tracking system operating at a sampling rate of 1000 Hz (SR Research, Ottawa, ON, Canada). The screen had a refresh rate of 144 Hz and a resolution of 1920 × 1080 pixels. The recording was monocular (from the right eye). The distance between the participant’s eyes and the screen was 93 cm, calculated to subtend approximately 1° of visual angle per character, given the sentences were presented in Courier New font size 25.

For the reading task (see Figure 1), a three-point calibration was performed prior to the start. Each trial began with a central fixation cross for 6000 ms, followed by the sentence presentation. Sentences were displayed individually, and participants read in a self-paced manner. A fixation point appeared on the left side of the screen for 500 ms before each sentence. Participants pressed a button to advance to the next sentence; if no response was detected within 8000 ms, the screen advanced automatically. Comprehension was assessed via 10 multiple-choice questions after each article. Practice trials were provided prior to the formal experiment. Throughout the task, participants were required to read five articles. The presentation order of these articles was randomized across subjects.

### 2.4. Data Analysis

This study selected reading comprehension accuracy and fixation duration measures as dependent variables. Reading comprehension accuracy served as an offline behavioral index, while fixation duration measures served as online behavioral indices reflecting real-time lexical processing. First, passages with reading comprehension accuracy below 60% were considered insufficiently processed by participants. Based on this criterion, accuracy and eye-movement data from these passages were excluded, accounting for 9.69% of the total reading passages. For eye-movement analysis at the lexical level, each word in the text was defined as an area of interest.

Following the criteria adopted in previous studies [10,50,51,52,53], raw data were processed in two steps. First, fixations shorter than 50 ms or longer than 1500 ms were excluded. Subsequently, further data was removed based on the following criteria: (1) loss of eye-tracking signal (2.63%); (2) durations exceeding three standard deviations from the mean (1.16%); and (3) fixations on the first and last words of each sentence (17.30%).

Reading comprehension accuracy was analyzed using analysis of variance (ANOVA). Fixation duration measures were analyzed using linear mixed-effects models (LMMs) implemented in the lme4 package [54] in R (version 4.5.1) [55]. All fixation duration measures were log-transformed before statistical analysis. In each model, the TMS-modulated brain region was included as a fixed effect, whereas participants and sentence trials were included as random effects. Model specification followed the principle of a maximal random-effects structure; when a model failed to converge, random effects were reduced stepwise until successful model convergence was achieved.

## 3. Results

The reading comprehension accuracy was 82.73% for the LIPL group, 83.77% for the CAL group, and 83.59% for the control group. A one-way ANOVA revealed no significant differences in reading comprehension accuracy among the three groups, *F*(2, 94) = 0.145, *p* = 0.866, *η*^2^ = 0.003.

**Figure 2:**
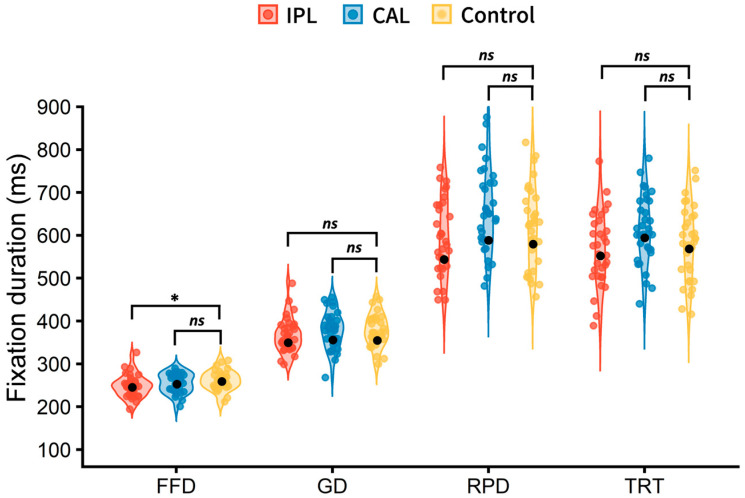
Fixation duration for the CAL group, LIPL group, and control group. FFD = first fixation duration; GD = gaze duration; RPD = regression path reading time; TRT = total reading time. The Control group received TMS over the vertex. The black dots represent the group means. * *p* < 0.05. *ns* = not significant (*p* > 0.05).

The eye-movement results are presented in Figure 2. Compared with the control group (262 ± 23 ms), the LIPL group (248 ± 27 ms) showed a significantly shorter FFD (*t* = −2.037, *p* = 0.045). However, no significant differences were observed between the LIPL and control groups for GD, RPD, or TRT (|*t*s| < 1.490, *p*s > 0.140). In contrast, the CAL group (255 ± 23 ms) did not show any significant differences compared to the control group in FFD (*t* = −0.918, *p* = 0.361). Similarly, no significant effects were found for the CAL group across other late processing measures, including GD, RPD, and TRT (|*t*s| < 1.384, *p*s > 0.170). The fixed-effect estimates of TMS modulation on fixation times across different brain regions are shown in Table 2.

## 4. Discussion

In this study, we employed TMS combined with eye-tracking to investigate the causal roles of the LIPL and CAL in natural L2 reading among Chinese–English bilinguals. The results showed that, relative to the control group, LIPL stimulation significantly reduced FFD, while producing no significant effects on GD, RPD, or TRT. Meanwhile, CAL stimulation yielded no significant effects on any of the eye-movement measures. These findings indicate that the LIPL plays a stage-specific causal role in L2 reading: TMS modulation of the LIPL facilitated early lexical recognition during English reading in Chinese–English bilinguals. This suggests that the LIPL may contribute to L2 reading by supporting early processing likely involving orthography-to-phonology conversion, providing stage-specific causal evidence for the involvement of the LIPL in English language processing among Chinese–English bilinguals.

Compared with the control group, TMS to the LIPL significantly reduced FFD. The LIPL is considered a key region for orthography-to-phonology conversion [23,56]. The meta-analysis by Li et al. [8] showed that L2 readers typically exhibit stronger LIPL activation than L1 readers during reading, reflecting the greater demands of phonological recoding when reading in a non-native language. Zhang et al. [9] further confirmed a functional preference of the LIPL for L2 reading. Costanzo et al. [57] found that TMS to the LIPL significantly improved accuracy in pseudoword reading but not in regular word reading, demonstrating the region’s specific sensitivity to phonological computation in alphabetic scripts. At the same time, the LIPL is also implicated in attention, working memory, and multimodal integration [58,59,60]. While cTBS is typically regarded as an inhibitory protocol, research has demonstrated that various protocol-related factors can substantially alter the direction and magnitude of cTBS aftereffects [34,61,62,63]. For example, Harrington et al. [34] found that cTBS over the supramarginal gyrus did not impair performance in a pseudoword discrimination task, but instead facilitated phonological processing speed. The authors interpreted this behavioral pattern as suggesting an excitatory response following cTBS in the reading system. Therefore, in the present study, we are inclined to interpret the reduced FFD as reflecting a facilitatory effect of cTBS over the LIPL, potentially through increased excitability of LIPL-related neural populations. One possible neurophysiological basis for this facilitatory effect is that cTBS may have transiently modulated the excitatory–inhibitory balance, potentially involving NMDA- and GABA-related processes [37,38,39]. FFD reflects early orthographic encoding and serves as an index of early lexical recognition efficiency [14]. Collectively, the reduction in FFD following LIPL modulation suggests that the LIPL contributes to early lexical recognition by facilitating orthography-to-phonology conversion and broader cognitive processes, thereby enhancing processing efficiency at this stage of L2 reading. However, given the absence of concurrent neurophysiological or neuroimaging measures, other possibilities, such as disinhibition of connected regions or transient network-level reorganization, cannot be entirely ruled out. It should also be acknowledged that the present behavioral data alone cannot fully determine the precise neurophysiological mechanism underlying this effect. Future studies in which participants perform the reading task during fMRI scanning after receiving TMS would help clarify the neural activity underlying the observed behavioral facilitation.

TMS to the LIPL did not significantly modulate GD, RPD, or TRT relative to the control group. These measures each capture, to varying degrees, reprocessing and integration beyond initial lexical access: GD reflects the sum of all first-pass fixation durations within a word region, including re-fixations and local reprocessing, while RPD and TRT are more closely associated with later-stage processes such as semantic integration and syntactic analysis [14]. Such later-stage processes might primarily be supported by regions outside the LIPL, including the left IFG and posterior MTG, which are implicated in unification operations and lexical-semantic retrieval during sentence comprehension [64,65,66]. As the LIPL’s functional contribution, particularly via its SMG subregion, is more closely tied to early phonological decoding than to semantic or syntactic integration, its modulation would not be expected to extend to these measures [67]. In contrast, FFD records only the first fixation on a word and is the most sensitive index of early lexical recognition [14]. Calvo and Meseguer [68] found that word frequency selectively influenced fixation duration at an early processing stage, further supporting the view that FFD specifically captures early, local lexical processing. Converging evidence from Sliwinska et al. [69] shows that TMS to the left SMG selectively disrupted phonological judgment while leaving semantic processing unaffected, confirming a content-specific causal role of this subregion. Collectively, the selective reduction in FFD with no significant effects on other measures suggests that the LIPL’s causal contribution to L2 reading is specific to the earliest stage of lexical recognition.

More broadly, the effects of brain stimulation on reading should also be understood from a state- and network-dependent perspective. Recent studies have suggested that cortical excitability may act as a state-sensitive factor influencing individual responsiveness to brain stimulation, and that focal stimulation may affect behavior through broader network-level modulation rather than isolated local effects alone [70,71]. In the present study, the LIPL was targeted as a reading-related region, but its contribution to natural L2 reading should be considered within the broader reading network. During natural L2 reading, early lexical processing is supported by coordinated activity across distributed reading-related regions. Thus, LIPL stimulation may have transiently modulated early-stage processing within this network, providing a more comprehensive understanding of the possible role of the LIPL in natural L2 reading.

TMS to the CAL produced no significant effects on any eye-movement measures. This null result may reflect two possibilities. First, the CAL may not be causally involved in L2 reading, and its activation observed in neuroimaging studies may be epiphenomenal rather than functionally necessary. Second, TMS may not have effectively engaged the target region. Participants generally did not report phosphene perception following stimulation, suggesting insufficient primary visual cortex activation [72]. The stimulation coordinate (x = −12, y = −84, z = 0), derived from the peak voxel of a voxel-wise brain-behavior correlation between calcarine activation and TRT in a simultaneous eye-tracking and fMRI study of natural English reading [18], presents two anatomical challenges: the CAL lies at considerable depth from the scalp surface, and the coordinate falls near the boundary between the CAL and the lingual gyrus, both of which may have limited the precision and depth of neuromodulation [73]. The causal role of the CAL in L2 reading remains to be clarified in future research, potentially employing neuromodulation techniques with greater penetration depth.

There were no significant differences between groups in reading comprehension accuracy. Reading comprehension accuracy was approximately 83% across all three groups (LIPL, CAL, and vertex control). This result admits two possible interpretations. First, TMS modulation of the target regions may not have affected overall L2 reading comprehension, which is consistent with the eye-movement findings in that TMS effects were primarily observed at the stage of early lexical recognition rather than at the level of higher-order text comprehension. Second, the uniformly high accuracy across groups suggests that the task may have been relatively easy, potentially introducing a ceiling effect that obscured any underlying group differences. This pattern is consistent with previous findings. Taylor et al. [74] found that intermittent theta burst stimulation (iTBS) to the dorsolateral prefrontal cortex significantly reduced frontoparietal network activation during a working memory task, yet produced no corresponding changes in behavioral accuracy or reaction time. Therefore, the comprehension accuracy measure was of limited sensitivity to TMS effects in the present study. Future research could increase task difficulty or adopt more sensitive measures of comprehension to more adequately capture the influence of TMS modulation on L2 reading comprehension.

Several methodological limitations and future directions should be noted. In terms of experimental operation, we used two cTBS applications within the same experimental session to help maintain the neuromodulatory effect across the relatively long natural reading task. This aspect of the design requires careful consideration because repeated cTBS may produce cumulative or potentially non-linear effects. Previous evidence suggests that the effects of repeated TBS depend on the interval between stimulation trains [45,46]. For cTBS, Gamboa et al. [45] found that delivering a second cTBS train after a 20 min interval extended the duration of the after-effects, and that the effects observed during this extended period were comparable to those induced by the first cTBS application. Our two cTBS applications were separated by approximately 20–30 min, which is broadly comparable to this interval. Nevertheless, because evidence on repeated cTBS in natural reading tasks remains limited, this procedure may have increased design complexity and inter-individual variability. Future studies should further examine repeated cTBS protocols in reading research. Regarding the stimulation protocol, future studies could also adopt a pre-post stimulation design to provide a more direct baseline for evaluating TMS-induced changes. Although the three groups in the present study were comparable in English proficiency, as indexed by CET-4 scores, Oxford Placement Test scores, and L2 self-ratings, reading performance was not measured before TMS. Future research could record reading comprehension accuracy and eye-movement measures both before and after stimulation. Such a stimulation site × session design would allow stronger within-subject comparisons, better control for individual differences in baseline reading performance, and potentially increase statistical power. Moreover, TMS aftereffects are relatively transient and time-dependent, typically lasting for 30–50 min depending on the stimulation protocol [44]. Thus, testing several hours after stimulation may yield different or weaker effects. Future research should further examine the temporal dynamics of TMS aftereffects in reading-related cognitive regions.

In summary, the present study found that, compared with the control group, TMS to the LIPL significantly enhanced early-stage processing efficiency in L2 reading, providing evidence for a stage-specific causal contribution of the LIPL to L2 reading. In contrast, TMS to the CAL produced no significant effects on any eye-movement measures, which may be attributable to the anatomical depth of the CAL rendering precise TMS modulation difficult. The causal role of CAL in L2 reading therefore remains to be further investigated.

## 5. Conclusions

In the present study, we employed TMS combined with eye-tracking to investigate the causal roles of the LIPL and the CAL in natural English reading among Chinese–English bilinguals. TMS to the LIPL significantly reduced FFD, indicating that the LIPL causally contributes to early lexical recognition in L2 reading in a stage-specific manner. TMS over the CAL produced no significant effects on any eye-movement measures, and its causal role remains to be further investigated. Overall, the present study provides evidence for a stage-specific causal role of the LIPL in L2 reading, and offers a theoretical basis for future neuromodulation-based interventions targeting early-stage L2 reading.

## Figures and Tables

**Table 1:** Backgrounds for participants in the Experiment.

Characteristics	LIPL Group	CAL Group	Control Group
*n*	32	33	32
Age (years)	21.59 (2.15)	21.85 (2.82)	20.75 (1.68)
L2 onset time (years)	7.38 (2.04)	8.03 (1.87)	7.70 (1.82)
CET-4 scores	511.97 (45.22)	505.45 (39.17)	514.16 (49.71)
OPT scores	35.19 (5.43)	33.97 (4.99)	35.28 (4.84)
L1 self-rating scores	8.62 (1.08)	8.94 (0.94)	8.98 (1.03)
L2 self-rating scores	6.25 (1.48)	6.16 (1.41)	6.47 (1.26)

Notes. L2 refers to second language. All stimulation targets are reported in Montreal Neurological Institute (MNI) coordinates. LIPL refers to the left inferior parietal lobule (x = −40, y = −38, z = 48 [8]). CAL refers to the calcarine cortex (x = −12, y = −84, z = 0 [18]). The control group received TMS over the vertex (x = 0, y = 0, z = 80 [41]). Standard deviations (SDs) are shown in parentheses. All participant characteristics were comparable across the LIPL, CAL, and control groups, with no significant differences between the experimental groups (LIPL and CAL) and the control group (all *p*s > 0.132).

**Table 2:** Fixed-effect estimates of TMS modulation on fixation times across different brain regions.

Fixation Times		*b*	*SE*	*t*	*p*	95% CI
First Fixation Duration	intercept	5.419	0.079	68.650	< 0.001	[5.264, 5.574]
	**LIPL vs. Vertex**	**−0.054**	**0.027**	**−2.037**	**0.045**	**[−0.106, −0.002]**
	CAL vs. Vertex	−0.024	0.027	−0.918	0.361	[−0.076, 0.028]
Gaze Duration	intercept	5.686	0.081	70.599	< 0.001	[5.528, 5.844]
	LIPL vs. Vertex	−0.036	0.027	−1.325	0.188	[−0.088, 0.017]
	CAL vs. Vertex	−0.002	0.027	−0.093	0.926	[−0.055, 0.050]
Regression Path Reading Time	intercept	6.128	0.096	63.529	< 0.001	[5.939, 6.317]
	LIPL vs. Vertex	−0.048	0.032	−1.490	0.140	[−0.110, 0.015]
	CAL vs. Vertex	0.008	0.032	0.256	0.799	[−0.054, 0.071]
Total Reading Time	intercept	6.056	0.111	54.772	< 0.001	[5.839, 6.273]
	LIPL vs. Vertex	−0.029	0.037	−0.779	0.438	[−0.101, 0.044]
	CAL vs. Vertex	0.051	0.037	1.384	0.170	[−0.021, 0.123]

Notes. LIPL = the left inferior parietal lobule; CAL = the calcarine cortex; *b* = raw (unstandardized) coefficient; *SE* = standard error; CI = confidence interval. The vertex stimulation group served as the control group. Brain region was dummy coded, with the vertex set as the baseline [0, 0], while the inferior parietal lobule was coded as [0, 1], and the calcarine cortex as [1, 0]. The bolded data indicate a significant difference (*p*s < 0.05) when compared to the vertex.

## Data Availability

The original data presented in the study are openly available at OSF: https://osf.io/g3kzs (accessed on 14 May 2026).

## Abbreviations

The following abbreviations are used in this manuscript:

L2	second language
L1	first language
CAL	calcarine cortex
LIPL	left inferior parietal lobule
TMS	transcranial magnetic stimulation
NMDA	N-methyl-D-aspartate
GABA	gamma-aminobutyric acid
FFD	first fixation duration
GD	gaze duration
RPD	regression path duration
TRT	total reading time
fMRI	functional magnetic resonance imaging
STG	superior temporal gyrus
IFG	inferior frontal gyrus
MNI	Montreal Neurological Institute
STEM	science, technology, engineering, and mathematics
MRI	magnetic resonance imaging
RMT	resting motor threshold
cTBS	continuous theta burst stimulation
LMM(s)	linear mixed-effects model(s)
MTG	middle temporal gyrus
SMG	supramarginal gyrus
iTBS	intermittent theta burst stimulation

## References

[1] Linck J.A., Osthus P., Koeth J.T., Bunting M.F. (2014). Working Memory and Second Language Comprehension and Production: A Meta-Analysis. Psychon. Bull. Rev..

[2] Cop U., Drieghe D., Duyck W. (2015). Eye Movement Patterns in Natural Reading: A Comparison of Monolingual and Bilingual Reading of a Novel. PLoS ONE.

[3] Kuperman V., Siegelman N., Schroeder S., Acartürk C., Alexeeva S., Amenta S., Bertram R., Bonandrini R., Brysbaert M., Chernova D. (2023). Text Reading in English as a Second Language: Evidence from the Multilingual Eye-Movements Corpus. Stud. Second Lang. Acquis..

[4] Price C.J. (2012). A Review and Synthesis of the First 20 years of PET and fMRI Studies of Heard Speech, Spoken Language and Reading. NeuroImage.

[5] Wu C.-Y., Ho M.-H.R., Chen S.-H.A. (2012). A Meta-Analysis of fMRI Studies on Chinese Orthographic, Phonological, and Semantic Processing. NeuroImage.

[6] Zhang G., Yuan B., Hua H., Lou Y., Lin N., Li X. (2021). Individual Differences in First-Pass Fixation Duration in Reading Are Related to Resting-State Functional Connectivity. Brain Lang..

[7] Henderson J.M., Choi W., Luke S.G. (2014). Morphology of Primary Visual Cortex Predicts Individual Differences in Fixation Duration during Text Reading. J. Cogn. Neurosci..

[8] Li H., Zhang J., Ding G. (2021). Reading across Writing Systems: A Meta-Analysis of the Neural Correlates for First and Second Language Reading. Bilingualism.

[9] Zhang J., Li H., Zhang M., Wang Z., Ao X., Jian J., Wei N., Liu H., Ding G., Meng X. (2023). Functional Preference of the Left Inferior Parietal Lobule to Second Language Reading. NeuroImage.

[10] Rayner K. (1998). Eye Movements in Reading and Information Processing: 20 Years of Research. Psychol. Bull..

[11] Bai X., Yan G., Liversedge S.P., Zang C., Rayner K. (2008). Reading Spaced and Unspaced Chinese Text: Evidence from Eye Movements. J. Exp. Psychol. Hum. Percept. Perform..

[12] Rayner K. (2009). The 35th Sir Frederick Bartlett Lecture: Eye Movements and Attention in Reading, Scene Perception, and Visual Search. Q. J. Exp. Psychol..

[13] Reichle E.D., Liversedge S.P., Drieghe D., Blythe H.I., Joseph H.S.S.L., White S.J., Rayner K. (2013). Using E-Z Reader to Examine the Concurrent Development of Eye-Movement Control and Reading Skill. Dev. Rev..

[14] Yan G., Xiong J., Zang C., Yu L., Cui L., Bai X. (2013). Review of Eye-movement Measures in Reading Research. Adv. Psychol. Sci..

[15] Weiss A.F. (2020). The Information Gathering Framework—A Cognitive Model of Regressive Eye Movements during Reading. J. Eye Mov. Res..

[16] Zhang J., Chen J., Ding G. (2024). Universality and Language Specificity of Brain Reading Networks: A Developmental Perspective. Dev. Sci..

[17] Wandell B.A., Rauschecker A.M., Yeatman J.D. (2012). Learning to See Words. Annu. Rev. Psychol..

[18] Wu J., Xin P., Pu X., Yan G., Gu C., Li H. (2026). Proficient Foreign Language Learners Resemble the Natives, Less Proficient Learner Struggles in Their Own Distinct Way: Evidence from Aligned Eye-Tracking and fMRI. J. Psycholinguist. Res..

[19] Chee M.W.L., Tan E.W.L., Thiel T. (1999). Mandarin and English Single Word Processing Studied with Functional Magnetic Resonance Imaging. J. Neurosci..

[20] Comstock L., Oliver B. (2021). A Meta-Analysis of Task-Based Differences in Bilingual L1 and L2 Language Networks. bioRxiv.

[21] Tan L.H., Laird A.R., Li K., Fox P.T. (2005). Neuroanatomical Correlates of Phonological Processing of Chinese Characters and Alphabetic Words: A Meta-analysis. Hum. Brain Mapp..

[22] Kim S.Y., Liu L., Cao F. (2017). How Does First Language (L1) Influence Second Language (L2) Reading in the Brain? Evidence from Korean-English and Chinese-English Bilinguals. Brain Lang..

[23] Golestani N., Pallier C. (2006). Anatomical Correlates of Foreign Speech Sound Production. Cereb. Cortex.

[24] Barbeau E.B., Chai X.J., Chen J.-K., Soles J., Berken J., Baum S., Watkins K.E., Klein D. (2017). The Role of the Left Inferior Parietal Lobule in Second Language Learning: An Intensive Language Training fMRI Study. Neuropsychologia.

[25] Pötzl O. (1925). Über Die Parietal Bedingte Aphasie Und Ihren Einfluss Auf Das Sprechen Mehrer Sprachen. Z. Gesamte Neurol. Psychiatr..

[26] Mechelli A., Crinion J.T., Noppeney U., O’Doherty J., Ashburner J., Frackowiak R.S., Price C.J. (2004). Structural Plasticity in the Bilingual Brain. Nature.

[27] Arrington C.N., Ossowski A.E., Baig H., Persichetti E., Morris R. (2023). The Impact of Transcranial Magnetic Stimulation on Reading Processes: A Systematic Review. Neuropsychol. Rev..

[28] Branzi F.M., Pobric G., Jung J., Lambon Ralph M.A. (2021). The Left Angular Gyrus Is Causally Involved in Context-Dependent Integration and Associative Encoding during Narrative Reading. J. Cogn. Neurosci..

[29] Ntemou E., Svaldi C., Jonkers R., Picht T., Rofes A. (2023). Verb and Sentence Processing with TMS: A Systematic Review and Meta-Analysis. Cortex.

[30] Qu X., Wang Z., Cheng Y., Xue Q., Li Z., Li L., Feng L., Hartwigsen G., Chen L. (2022). Neuromodulatory Effects of Transcranial Magnetic Stimulation on Language Performance in Healthy Participants: Systematic Review and Meta-Analysis. Front. Hum. Neurosci..

[31] Turker S., Kuhnke P., Schmid F.R., Cheung V.K.M., Weise K., Knoke M., Zeidler B., Seidel K., Eckert L., Hartwigsen G. (2023). Adaptive Short-Term Plasticity in the Typical Reading Network. NeuroImage.

[32] Acheson D.J., Hagoort P. (2013). Stimulating the Brain’s Language Network: Syntactic Ambiguity Resolution after TMS to the Inferior Frontal Gyrus and Middle Temporal Gyrus. J. Cogn. Neurosci..

[33] Terranova C., Rizzo V., Cacciola A., Chillemi G., Calamuneri A., Milardi D., Quartarone A. (2019). Is There a Future for Non-Invasive Brain Stimulation as a Therapeutic Tool?. Front. Neurol..

[34] Harrington R.M., Krishnamurthy L.C., Ossowski A., Jeter M., Davis A., Bledniak E., Ware A.L., Morris R., Arrington C.N. (2023). Preliminary Evidence of Prolonged Timing Effects of Theta-Burst Stimulation in the Reading System. Front. Hum. Neurosci..

[35] Rossi S., Hallett M., Rossini P.M., Pascual-Leone A. (2009). Safety, Ethical Considerations, and Application Guidelines for the Use of Transcranial Magnetic Stimulation in Clinical Practice and Research. Clin. Neurophysiol..

[36] Rossi S., Antal A., Bestmann S., Bikson M., Brewer C., Brockmöller J., Carpenter L.L., Cincotta M., Chen R., Daskalakis J.D. (2021). Safety and Recommendations for TMS Use in Healthy Subjects and Patient Populations, with Updates on Training, Ethical and Regulatory Issues: Expert Guidelines. Clin. Neurophysiol..

[37] Matsuta H., Shimomura T., Kouchiyama T., Fujiki M. (2022). Continuous Theta-Burst Stimulation to the Sensorimotor Cortex Affects Contralateral Gamma-Aminobutyric Acid Level and Resting-State Networks. PLoS ONE.

[38] Stagg C.J., Wylezinska M., Matthews P.M., Johansen-Berg H., Jezzard P., Rothwell J.C., Bestmann S. (2009). Neurochemical Effects of Theta Burst Stimulation as Assessed by Magnetic Resonance Spectroscopy. J. Neurophysiol..

[39] Huang Y.-Z., Chen R.-S., Rothwell J.C., Wen H.-Y. (2007). The After-Effect of Human Theta Burst Stimulation Is NMDA Receptor Dependent. Clin. Neurophysiol..

[40] Wu J., Ji Y., Qu H., Zuo S., Liang J., Su J., Wang Q., Yan G., Ding G. (2025). Transcranial Magnetic Stimulation of the Right Inferior Frontal Gyrus Impairs Bilinguals’ Performance in Language-Switching Tasks. Cognition.

[41] Jung J., Bungert A., Bowtell R., Jackson S.R. (2016). Vertex Stimulation as a Control Site for Transcranial Magnetic Stimulation: A Concurrent TMS/fMRI Study. Brain Stimul..

[42] Follmer D.J., Fang S.-Y., Clariana R.B., Meyer B.J.F., Li P. (2018). What Predicts Adult Readers’ Understanding of STEM Texts?. Read. Writ..

[43] Ma X., Liu Y., Clariana R., Gu C., Li P. (2022). From Eye Movements to Scanpath Networks: A Method for Studying Individual Differences in Expository Text Reading. Behav. Res..

[44] Huang Y.-Z., Edwards M.J., Rounis E., Bhatia K.P., Rothwell J.C. (2005). Theta Burst Stimulation of the Human Motor Cortex. Neuron.

[45] Gamboa O.L., Antal A., Laczo B., Moliadze V., Nitsche M.A., Paulus W. (2011). Impact of Repetitive Theta Burst Stimulation on Motor Cortex Excitability. Brain Stimul..

[46] Nyffeler T., Wurtz P., Lüscher H., Hess C.W., Senn W., Pflugshaupt T., Von Wartburg R., Lüthi M., Müri R.M. (2006). Extending Lifetime of Plastic Changes in the Human Brain. Eur. J. Neurosci..

[47] Lissemore J.I., Buchanan D.M., Batail J.-M., Kaloiani I., Veerapal C., Sahlem G.L., Williams N.R. (2024). Strategies to Mitigate Scalp Discomfort during Repetitive Transcranial Magnetic Stimulation. Brain Stimul..

[48] Loo C.K., McFarquhar T.F., Mitchell P.B. (2008). A Review of the Safety of Repetitive Transcranial Magnetic Stimulation as a Clinical Treatment for Depression. Int. J. Neuropsychopharmacol..

[49] Borckardt J.J., Nahas Z.H., Teal J., Lisanby S.H., McDonald W.M., Avery D., Durkalski V., Pavlicova M., Long J.M., Sackeim H.A. (2013). The Painfulness of Active, but Not Sham, Transcranial Magnetic Stimulation Decreases Rapidly Over Time: Results From the Double-Blind Phase of the OPT-TMS Trial. Brain Stimul..

[50] Inhoff A.W., Briihl D., Schwartz J. (1996). Compound Word Effects Differ in Reading, on-Line Naming, and Delayed Naming Tasks. Mem. Cogn..

[51] Desai R.H., Choi W., Henderson J.M. (2020). Word Frequency Effects in Naturalistic Reading. Lang. Cogn. Neurosci..

[52] Zhou W., Wang S., Yan M. (2023). Fixation-Related fMRI Analysis Reveals the Neural Basis of Natural Reading of Unspaced and Spaced Chinese Sentences. Cereb. Cortex.

[53] Schuster S., Himmelstoss N.A., Hutzler F., Richlan F., Kronbichler M., Hawelka S. (2021). Cloze Enough? Hemodynamic Effects of Predictive Processing during Natural Reading. NeuroImage.

[54] Bates D., Mächler M., Bolker B., Walker S. (2015). Fitting Linear Mixed-Effects Models Using *Lme4*. J. Stat. Soft..

[55] R Core Team (2024). R: A Language and Environment for Statistical Computing.

[56] Sliwinska M.W. (2015). The Role of the Left Inferior Parietal Lobule in Reading. Ph.D. Thesis.

[57] Costanzo F., Menghini D., Caltagirone C., Oliveri M., Vicari S. (2012). High Frequency rTMS over the Left Parietal Lobule Increases Non-Word Reading Accuracy. Neuropsychologia.

[58] Caspers S., Schleicher A., Bacha-Trams M., Palomero-Gallagher N., Amunts K., Zilles K. (2013). Organization of the Human Inferior Parietal Lobule Based on Receptor Architectonics. Cereb. Cortex.

[59] Baldo J.V., Dronkers N.F. (2006). The Role of Inferior Parietal and Inferior Frontal Cortex in Working Memory. Neuropsychology.

[60] Numssen O., Bzdok D., Hartwigsen G. (2021). Functional Specialization within the Inferior Parietal Lobes across Cognitive Domains. eLife.

[61] Hamada M., Murase N., Hasan A., Balaratnam M., Rothwell J.C. (2013). The Role of Interneuron Networks in Driving Human Motor Cortical Plasticity. Cereb. Cortex.

[62] Vernet M., Bashir S., Yoo W.-K., Oberman L., Mizrahi I., Ifert-Miller F., Beck C.J., Pascual-Leone A. (2014). Reproducibility of the Effects of Theta Burst Stimulation on Motor Cortical Plasticity in Healthy Participants. Clin. Neurophysiol..

[63] Doeltgen S.H., Ridding M.C. (2011). Low-Intensity, Short-Interval Theta Burst Stimulation Modulates Excitatory but Not Inhibitory Motor Networks. Clin. Neurophysiol..

[64] Hagoort P. (2013). MUC (Memory, Unification, Control) and Beyond. Front. Psychol..

[65] Chen L., Wu J., Hartwigsen G., Li Z., Wang P., Feng L. (2021). The Role of a Critical Left Fronto-Temporal Network with Its Right-Hemispheric Homologue in Syntactic Learning Based on Word Category Information. J. Neurolinguist..

[66] Rivera M., Paolieri D., Iniesta A., Pérez A.I., Bajo T. (2023). Second Language Acquisition of Grammatical Rules: The Effects of Learning Condition, Rule Difficulty, and Executive Function. Bilingualism.

[67] Stoeckel C., Gough P.M., Watkins K.E., Devlin J.T. (2009). Supramarginal Gyrus Involvement in Visual Word Recognition. Cortex.

[68] Calvo M.G., Meseguer E. (2002). Eye Movements and Processing Stages in Reading: Relative Contribution of Visual, Lexical, and Contextual Factors. Span. J. Psychol..

[69] Sliwinska M.W., Khadilkar M., Campbell-Ratcliffe J., Quevenco F., Devlin J.T. (2012). Early and Sustained Supramarginal Gyrus Contributions to Phonological Processing. Front. Psychol..

[70] Di Fazio C., Scaliti E., Stanziano M., Nigri A., Demichelis G., Tamietto M., Palermo S. (2026). Exploring Cortical Excitability Modulation to Promote Cognitive Resilience in Aging: An rTMS Study Protocol. Front. Hum. Neurosci..

[71] Di Fazio C., Palermo S. (2026). A Prefrontal Neuromodulation Route for Post-Traumatic Olfactory Dysfunction: A Perspective Supported by Recovery During Left-DLPFC rTMS. Brain Sci..

[72] Cowey A., Walsh V. (2000). Magnetically Induced Phosphenes in Sighted, Blind and Blindsighted Observers. NeuroReport.

[73] Deng Z.-D., Lisanby S.H., Peterchev A.V. (2013). Electric Field Depth–Focality Tradeoff in Transcranial Magnetic Stimulation: Simulation Comparison of 50 Coil Designs. Brain Stimul..

[74] Taylor S.F., Gu P., Simmonite M., Lasagna C., Tso I.F., Lee T.G., Vesia M., Hernandez-Garcia L. (2024). Lateral Prefrontal Stimulation of Active Cortex with Theta Burst Transcranial Magnetic Stimulation Affects Subsequent Engagement of the Frontoparietal Network. Biol. Psychiatry Cogn. Neurosci. Neuroimaging.

